# Nano-Cavities within Nano-Zeolites: The Influencing Factors of the Fabricating Process on Their Catalytic Activities

**DOI:** 10.3390/nano13131923

**Published:** 2023-06-23

**Authors:** Kairui Fu, Geng Li, Fulin Xu, Tiantong Dai, Wen Su, Hao Wang, Tianduo Li, Yunan Wang, Jingui Wang

**Affiliations:** 1 School of Chemistry and Chemical Engineering, University of Jinan, Jinan 250022, China; kr475686761@163.com; 2 Shandong Provincial Key Laboratory of Fine Chemicals, School of Chemistry and Chemical Engineering, Qilu University of Technology (Shandong Academy of Sciences), Jinan 250353, China; iamligeng97@163.com (G.L.); xufulin97@163.com (F.X.); daitiantong0906@163.com (T.D.); woshisuwen@163.com (W.S.); wanghao6215@163.com (H.W.); 3 Key Laboratory of Advanced Fuel Cells and Electrolyzers Technology of Zhejiang Province, Ningbo Institute of Materials Technology and Engineering, Chinese Academy of Sciences, Ningbo 315201, China

**Keywords:** zeolites, nano-cavities, hollow, titanium silicalite-1, TS-1, oxidation

## Abstract

Titanium silicalite-1 (TS-1) is a milestone heterogeneous catalyst with single-atom tetrahedral titanium incorporated into silica framework for green oxidation reactions. Although TS-1 catalysts have been industrialized, the strategy of direct hydrothermal synthesis usually produces catalysts with low catalytic activities, which has still puzzled academic and industrial scientists. Post-treatment processes were widely chosen and were proven to be an essential process for the stable production of the high-activity zeolites with hollow structures. However, the reasons why post-treatment processes could improve catalytic activity are still not clear enough. Here, high-performance hollow TS-1 zeolites with nano-sized crystals and nano-sized cavities were synthesized via post-treatment of direct-synthesis nano-sized TS-1 zeolites. The influencing factors of the fabricating processes on their catalytic activities were investigated in detail, including the content of alkali metal ions, the state of titanium centers, hydrophilic/hydrophobic properties, and accessibility of micropores. The post-treatment processes could effectively solve these adverse effects to improve catalytic activity and to stabilize production. These findings contribute to the stable preparation of high-performance TS-1 catalysts in both factories and laboratories.

## 1. Introduction

Titanium silicalite-1 (TS-1) is a category of MFI-type microporous zeolite catalyst with single-atom tetrahedral titanium incorporated into the silica lattice, which can activate hydrogen peroxide to selectively oxidize alkanes, alkenes, ketones, phenols, etc. [1,2,3,4]. TS-1 zeolite was first commercially produced by Enichem in 1983 [5] and quickly became the core catalyst for green oxidation processes in the industry, including phenol hydroxylation, ketone ammoximation, and alkene epoxidation [6,7,8,9,10,11]. Although TS-1 catalysts have been industrialized by a rigorous process, a controlled synthetic strategy for the stable production of high-activity TS-1 zeolites still puzzles academic and industrial scientists. For example, self-made TS-1 samples characterized with similar physicochemical properties displayed quite different catalytic activities as reported in the literature. The TS-1 catalyst prepared by using a similar method but in different factories/laboratories also showed different catalytic activities. Regarding the bad production reproducibility, scientists have never stopped searching for the reasons and effective factors. Van der Pol and his co-workers found that it was impossible to differentiate active and less active samples by using XRD, IR, ^29^Si NMR, and DREAS measurements but particle size determination could explain these differences [12]. Smaller particles were more active than larger particles as the larger zeolite particles are not fully utilized in the internal pores attributed to the diffusion limitations. In addition, the adverse effects of alkali metal ions on the synthesis of TS-1 have been pointed out since the first reports [5]. It was revealed that the raw materials for preparing TS-1 catalysts should be free of alkali metal ions. The presence of small amounts of alkali metal ions in the preparation procedures would eliminate the catalytic activity of the obtained TS-1 [6,13]. Therefore, the alkali metal ions were thought to be an influence factor responsible for the bad reproducibility because of the inevitable contamination of alkali metal ions in the raw materials, especially in industrial processes. Davis et al. confirmed the adverse effects of alkali metal ions and found that the simple acid post-treatment was useful in overcoming the problems of alkali metal ions [14]. The reason was that the alkali metal ions in the micropores of the TS-1 molecular sieve could be washed off to recover the active titanium. The recovery intensity of activity was related to the content of alkali metal ions in the samples. Only TS-1 that contained very small amounts of alkali metal ions could be fully recovered. Our research group focused on the production of high activity TS-1 zeolites by the one-pot process using industrial-grade raw materials [15,16,17,18]. It was found that the presence of alkali metal ions in the synthetic system greatly affected the catalytic activity of TS-1 catalysts, resulting in low activity and bad reproducibility.

Significantly, Lin et al. from China Petroleum & Chemical Corporation (SINOPEC) developed a new synthetic strategy to stably prepare TS-1 catalysts with a hollow structure [19,20,21]. Post-treatment recrystallization was the key process [22]. In addition to increasing the catalytic activity, this strategy could stabilize production and reduce the risk of failure, which had become a widely-accepted essential process for the production of the TS-1 zeolite catalyst [23,24,25,26,27,28,29,30,31]. The post-treatment to enhance catalytic activity was explained from the aspects of reducing microporous channels based on a hollow structure and/or improving the content of active titanium [11,23,31,32]. In the following research, the studies were focused on the modification of post-treatment processes and the development of catalytic applications of hollow TS-1 [32,33,34,35,36,37,38,39,40,41]. The influencing factors why post-treatment could enhance catalytic activity, however, are still not clear enough.

Here, high-activity hollow TS-1 zeolites with nano-sized crystals and nano-sized cavities were stably produced via post-treatment of direct-synthesis TS-1 zeolites. The influencing factors, including the content of alkali metal ions, the state of titanium, hydrophilic/hydrophobic properties, and accessibility of micropores were investigated in each step of the post-treatment process through XRD, SEM, TEM, DRUV, FTIR, ICP-OES, nitrogen adsorption, and water adsorption characterizations. We tried to find out the key effective factors on catalytic activity in alkene epoxidation during the post-treatment processes, and ranked the importance of these effective factors, hoping that this could provide help for the stable production of high-activity TS-1 zeolite catalysts.

## 2. Materials and Methods

### 2.1. Materials

Titanium tetra-n-butoxide (reagent grade, >98.5%), tetraethyl orthosilicate (reagent grade, 98%), and tetrapropylammonium hydroxide (TPAOH, 1.0 mol/L in water; ICP testing showed the content K^+^ was 0.70 wt.% and the content Na^+^ was 0.035 wt.%) were bought from Sigma-Aldrich Company Ltd. Other reagents including H_2_SO_4_ (reagent grade, 98%), triethanolamine (reagent grade, 97%), hydrogen peroxide (30%), 1-hexene (reagent grade, 99%), cyclohexanone (reagent grade, 99%), and Ce(SO_4_)_2_ (standard solution, 0.1000 mol/L) were purchased from Macklin. All the chemical reagents were used without further purifications.

### 2.2. Syntheses of TS-1 Catalysts

The syntheses of parent TS-1 catalysts were according to the reported paper with modification [17]. Titanium tetra-n-butoxide used as a titanium source, tetraethyl orthosilicate as a silicon source, and tetrapropylammonium hydroxide (TPAOH) as an organic structure-directing agent were mixed under stirring at room temperature. The mixture with a molar composition of 1.0 SiO_2_:0.05 TiO_2_:0.45 TPAOH:35 H_2_O was transferred into an autoclave and treated at 443 K for 2 days. The solids were separated by centrifugation, washed with distilled water several times until the pH lower than 8.0, dried at 80 °C for 24 h, and then calcined at 823 K for 6 h, designated as TS-1-p.

### 2.3. Post-Treatment Procedures

The synthesized parent TS-1 zeolites were subjected to a two-step post-treatment, including acid washing and basic recrystallization. Acid washing post-treatment was conducted at 363 K for 5 h in a 0.05 mol/L H_2_SO_4_ aqueous solution with a liquid-to-solid (zeolite) ratio of 30 mL to 1 g. Basic recrystallization processes were conducted at 463 K for 12 h in an autoclave filled with zeolites and a mixed aqueous solution of 1.0 wt.% triethanolamine and 1.0 wt.% tetrapropylammonium hydroxide with a liquid-to-solid ratio of 30 mL to 1 g. The samples in each step were separated by centrifugation, washed with distilled water several times until the pH lower than 8.0, dried at 80 °C for 24 h, and calcination at 823 K for 6 h. Sample suffering from acid washing post-treatment was designated as TS-1-p-ac. The samples of acid post-treatment further suffering basic recrystallization post-treatment were designated as TS-1-p-ac-re.

### 2.4. Characterization

X-ray diffraction (XRD) was carried out on a Bruker Powder D8 Advance diffractometer with CuKα radiation (λ = 1.5418 Å). Field-emission scanning electron microscope (SEM) images were tested on a JEOL JSM-7600F microscope operated at 5 kV. Transmission electron microscopy (TEM) images were performed using a JEOL JEM-1400 TEM microscope working at 100 kV. The samples for TEM measurements were dispersed in ethanol ultrasonically, dropped on copper grids, and then dried at 373 K. Nitrogen adsorption–desorption characterization was evaluated on Quantachrome Autosorb-iQ instruments at 77 K liquid nitrogen. The samples were degassed at 573 K under vacuum for 10 h before testing. Diffuse reflectance ultraviolet–visible (DRUV/vis) spectra were recorded on a Shimadzu UV-2450 spectrophotometer with BaSO4 as a reference. Fourier Transform Infrared (FTIR) spectra were measured on a Shimadzu IRPrestige-21 spectrometer based on KBr pellets. Elemental analysis of Si, Ti, Na, and K was measured on a Perkin Elmer ICP Optima 2000DV (Waltham, MA, USA) inductively coupled plasma optical emission spectrometer.

### 2.5. Catalytic Testing

The model epoxidation reaction of 1-hexene was performed in a 20 mL glass reactor. Catalyst (25 mg), 1-hexene (5 mmol), H_2_O_2_ (30%, 5 mmol), and methanol solvent (5 mL) were added into the reactor. The mixture was reacted at 333 K with vigorous stirring. After the reaction, the mixture was analyzed by gas chromatography (Shimadzu GC-2014 equipped with a 30 m TC-1 capillary column and a flame ionization detector). The products were verified on a Shimadzu PARVUM 2 gas chromatograph mass spectrometer. Cyclohexanone as an internal standard was added after reaction to calculate the mass of the reactants and products. The remaining H_2_O_2_ was determined with a standard Ce(SO_4_)_2_ solution (0.1 mol/L) by titration.

## 3. Results and Discussion

**Figure 1:**
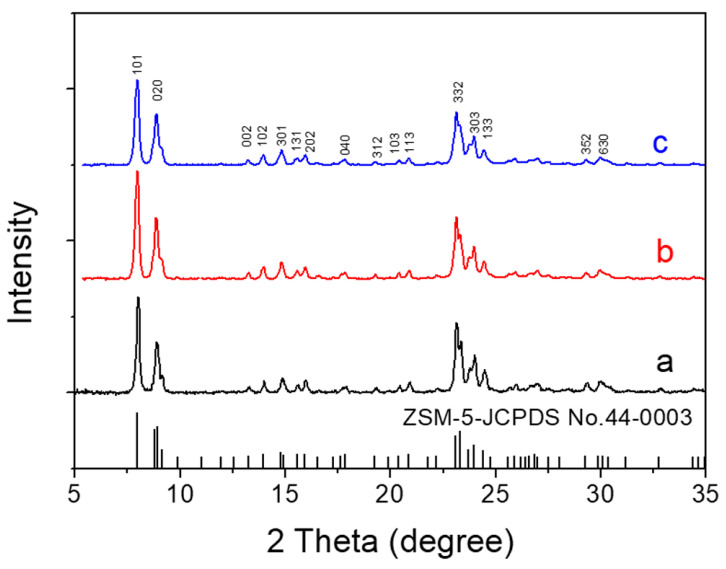
XRD patterns of TS-1 zeolites obtained in different steps. (a) Parent nano-zeolites via direct synthesis, (b) after acid washing post-treatment, and (c) after further basic recrystallization post-treatment.

XRD patterns (Figure 1) displayed that all the zeolites obtained from different steps had a pure MFI structure, indicating that the post-treatment process did not lead to the collapse of the TS-1 crystalline structure.

**Figure 2:**
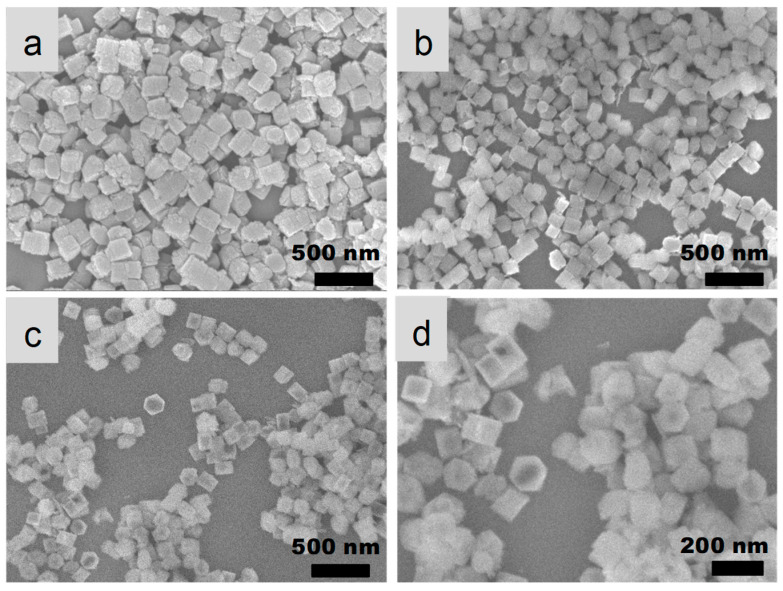
SEM images of TS-1 zeolites obtained in different steps. (**a**) Parent nano-zeolites via direct synthesis, (**b**) after acid washing post-treatment, (**c**) after further basic recrystallization post-treatment, and (**d**) enlarged image for sample after further basic recrystallization post-treatment.

SEM images (Figure 2) revealed that the particle size of parent zeolites was about 200–300 nm. The morphology had no change after acid washing post-treatment. It was found that some tiny materials were present on the surface of zeolitic crystals. After recrystallization treatment, the size of the crystal did not change obviously, but the edges and corners of the crystal were much clearer. In addition, the crystal surface was also clean. The results suggested that the recrystallization post-treatment processes did lead to the dissolution and regrowth of TS-1 crystals.

**Figure 3:**
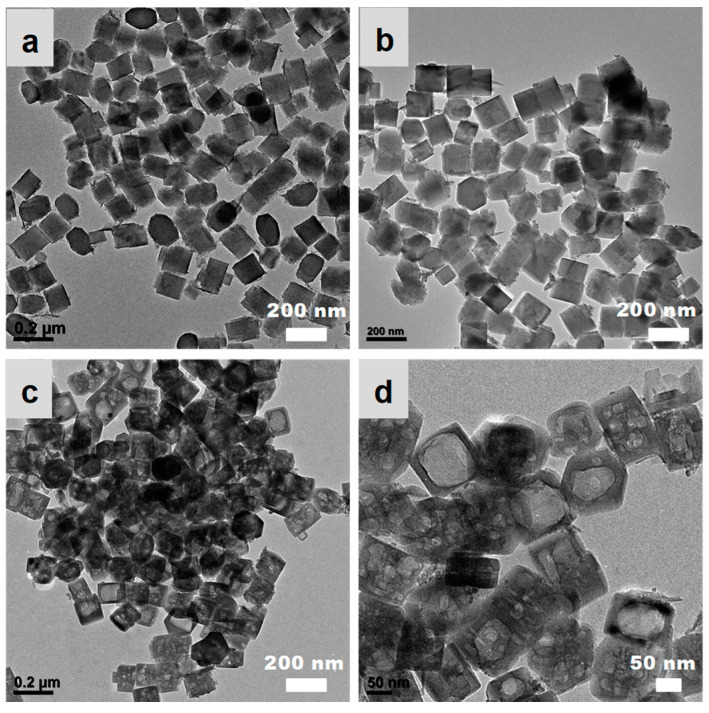
TEM images of TS-1 zeolites obtained in different steps. (**a**) Parent nano-zeolites via direct synthesis, (**b**) after acid washing post-treatment, (**c**) after further basic recrystallization post-treatment, and (**d**) enlarged image for sample after further basic recrystallization post-treatment.

TEM images (Figure 3) indicated the morphology changed little after acid washing treatment. It was found some tiny nanoparticles and plate-like nanomaterials were present on the crystal surface. After the basic recrystallization processes, the obvious hollow structure with one or more nano-cavities (5–100 nm) in one zeolitic crystal and clear crystal edges were formed, suggesting the recrystallization processes were accompanied by the processes of internal dissolution and shell reorganization. This phenomenon was consistent with the previously reported alkali treatment processes of zeolites, during which the interior first dissolved to form a box-like hollow structure because of the different cross-linkage degree of the inner part and outer shell [42,43]. In addition, the internal dissolved cavities had certain geometric shapes. The hexagonal cavity morphology could be clearly seen in some areas. This may be related to the intrinsic mosaic-like stacking structure of zeolite crystals [44,45].

**Figure 4:**
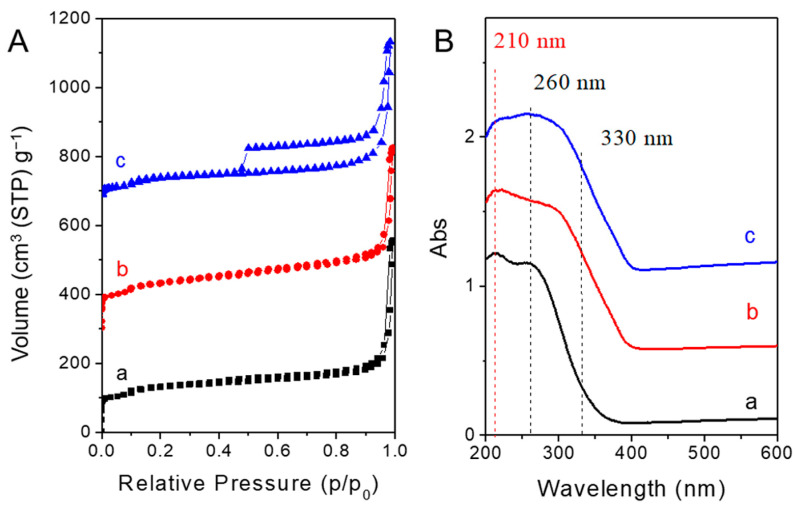
(**A**) Nitrogen adsorption–desorption isotherms and (**B**) DRUV/vis spectra of TS-1 zeolites obtained in different steps. (a) Parent nano-zeolites via direct synthesis, (b) after acid washing post-treatment, and (c) after further basic recrystallization post-treatment.

Nitrogen adsorption–desorption isotherms (Figure 4A) showed that parent nano-zeolites via direct synthesis were a typical type-I microporous adsorption isotherm, and there was almost no change during the acid post-treatment. After recrystallization, an apparent H4 hysteresis loop between the adsorption and desorption isotherm was observed, indicating the hollow structure [8]. This was consistent with the structure observed by TEM. The BET surface areas and microporous volumes changed little after acid washing (Table 1). The increase in the external specific surface area after acid treatment may be related to the formation of a certain amount of nano-materials on the crystal surface, as shown in the SEM (Figure 2b) and TEM images (Figure 3b). After recrystallization post-treatment processes, the BET surface areas increased. This may be related to the formation of hollow structures. This was also reflected in the rising part at relatively high pressure (p/p_0_ > 0.9), which overlaps with the stacking pores between zeolite nano-particles. Due to the wide distribution of hollow sizes (5–100 nm), it is not possible to form a concentrated pore size distribution, which is why the pore size distribution curve cannot be obtained here. The decrease in the external specific surface area was attributed to the decrease of the nano-materials on the crystal surface and the slight increase of crystal size by a recrystallization.

Diffuse reflectance UV/vis (DRUV/vis) spectroscopy is a powerful technique to detect the states of titanium species in TS-1 [4]. The absorbance peaks at approximately 210 nm, 260 nm, and 330 nm in the DRUV/vis spectrum were assigned to the single-atom tetrahedral framework titanium species, isolated extra-framework titanium species, and tiny TiO_2_ particles, respectively. It has been proven that the framework Ti species provide a high catalytic activity. As shown in Figure 4B, the DRUV/vis spectra showed that parent TS-1 nano-zeolites via direct synthesis had significant amounts of extra-framework titanium species. Acid and recrystallization post-treatment led to an increase in extra-framework titanium species and the formation of a TiO_2_ phase. Therefore, from only the DRUV/vis spectrum characterizations, the post-treatment did not effectively improve the framework titanium species, but formed some titanium species that may be not beneficial to the catalytic activities.

**Figure 5:**
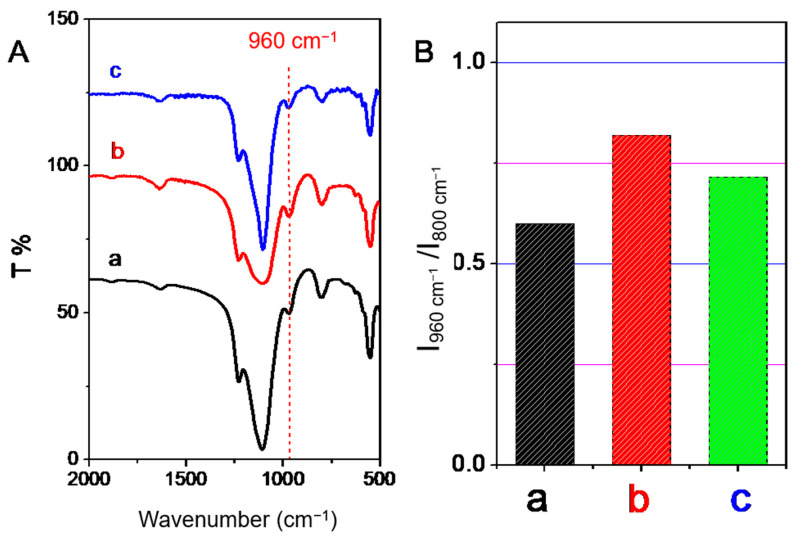
(**A**) FTIR spectra and (**B**) intensity ratio of 960 cm^−1^ to 800 cm^−1^ in the FTIR spectra of TS-1 zeolites obtained in different steps. (a) Parent nano-zeolites via direct synthesis (b) after acid washing post-treatment, and (c) after further basic recrystallization post-treatment.

As shown in Figure 5A, the absorption peak of 960 cm^−1^ in FTIR spectrum is considered as the signal peak of active framework titanium species [6,13]. Importantly, this band intensity proportionally increases with the increase of the amounts of the framework titanium species [46]. For an easy comparison, this characteristic peak was normalized based on the band at 800 cm^−1^ indexed as the symmetric Si-O-Si vibration, as shown in Figure 5B. After acid treatment, the peak centered at 960 cm^−1^ increased, and then decreased after recrystallization processes, which was still higher than that of the parent sample. It should be noted that the vibration absorption peak of Si-OH was also around 960 cm^−1^, which had a contribution to the peak of 960 cm^−1^ band [18]. Therefore, the reason for the improvement of the 960 cm^−1^ band by acid treatment may be that part of the framework titanium was leached as indicated in the UVDR/vis analysis to leave some Si-OH nests. During the recrystallization post-treatment processes, these silanols generated by the acid washing were recovered again with the migration of silicon species and/or titanium species, resulting in the reduction of the number of silanol groups. Moreover, the leaching of framework titanium to form extra-framework titanium species was observed by UVDR/vis analysis after the recrystallization post-treatment processes. Both of these aspects will lead to a decrease of 960 cm^−1^ peaks in FTIR spectra during the recrystallization post-treatment processes.

**Figure 6:**
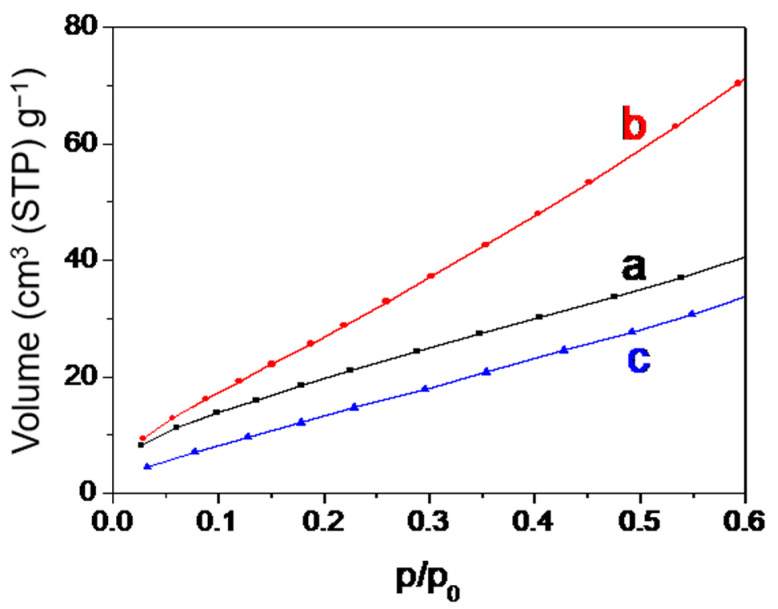
Water adsorption curves of TS-1 zeolites obtained in different steps. (a) Parent nano-zeolite via direct synthesis, (b) after acid washing post-treatment, and (c) after further basic re-crystallization post-treatment.

Many reports revealed that hydrophilic and hydrophobic properties had an important influence on catalytic performance of the TS-1 catalyst [47,48,49,50]. Usually, the better the hydrophobicity, the higher the catalytic performance in the alkene epoxidation reactions. As shown in Figure 6, water adsorption curves demonstrated that acid post-treatment improved the hydrophilicity, which may be related to the increase of silanol groups mentioned above. The recrystallization post-treatment processes effectively reduced the hydrophilicity of the catalyst, which was much lower than that of the parent sample. This suggested that the silanol defects/nests were filled/recovered by the condensation of silanols attributed to the migrated silicon and/or titanium during the recrystallization process, which led to the reduction of the amounts of silicon hydroxyl groups, thus reducing the hydrophilicity.

**Figure 7:**
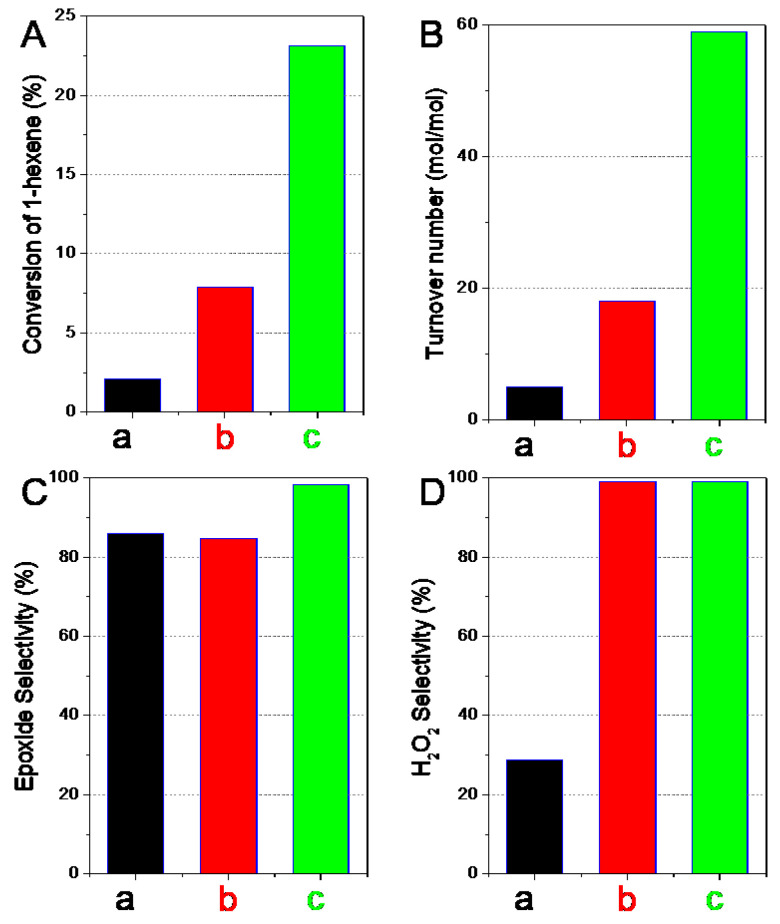
Catalytic performances including (**A**) conversion of 1-hexene, (**B**) turnover number, (**C**) epoxide selectivity, and (**D**) H_2_O_2_ selectivity of TS-1 zeolites obtained in different steps. (a) Parent nano-zeolite via direct synthesis, (b) after acid washing post-treatment, and (c) after further basic recrystallization post-treatment.

**Figure 8:**
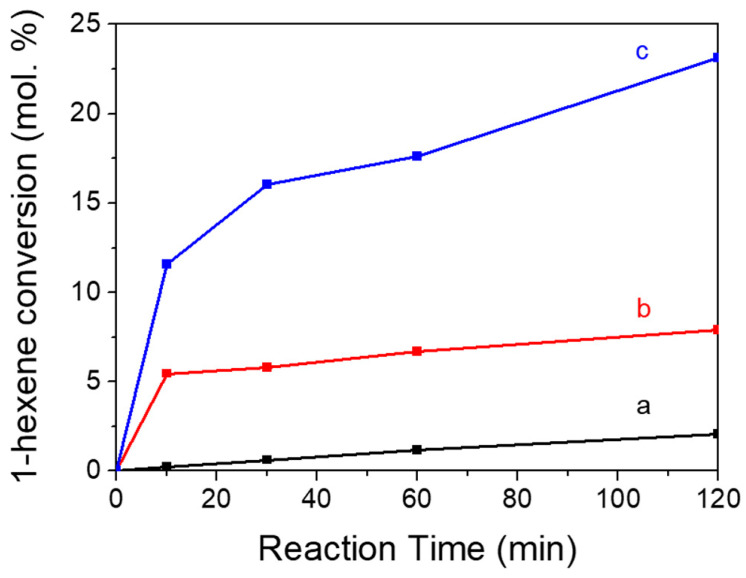
Time courses of catalytic reactions for 1-hexene oxidation with H_2_O_2_ using TS-1 catalysts obtained in different steps. (a) parent nano-zeolite via direct synthesis, (b) after acid washing post-treatment, and (c) after further basic recrystallization post-treatment.

A model reaction of 1-hexene epoxidation with H_2_O_2_ was chosen to evaluate the catalytic activity of TS-1 zeolite catalyst by post-treatments. The catalytic results are shown in Table 2, and Figure 7 and Figure 8.

The conversion of 1-hexene, turnover number, and the initial reaction rate increased after acid washing post-treatment. In addition, the selectivity in the use of H_2_O_2_ toward the epoxides and their derivatives greatly increased to close to 100%. After further basic recrystallization post-treatment, the conversion of 1-hexene was significantly improved to 23.1%; it had increased by about three times compared to sample after acid post-treatment. The catalytic results were slightly better than that of the reported TS-1 catalyst under similar catalytic reaction conditions [39,51,52]. In addition, the catalytic activity had achieved similar catalytic results as the commercial TS-1 product (24.4% as shown in Table 2). Importantly, the epoxide selectivity of 98.2% and H_2_O_2_ efficiency near 100% were achieved, which were both better than the commercial TS-1.

The effects of post-treatment on the catalytic performance were discussed combined with the previous characterization as follows:

(1) Acid treatment could eliminate alkali metal ions in the TS-1 catalyst, improving the catalytic activity and the utilization efficiency of hydrogen peroxide. Meanwhile, acid treatment resulted in the increase of silanol groups and the decrease of hydrophobicity as evaluated by water adsorption testing (Figure 6), which was not beneficial to catalytic activity. In addition, acid treatment led to the leaching of active framework titanium species to form extra-framework titanium species and/or anatase-like tiny TiO_2_ based on the increased adsorption band at about 260 nm and 330 nm in the DRUV/vis spectra (Figure 4B), which would have adverse effects on catalytic activity.
(2) The recrystallization post-treatment process could form nano-cavities within zeolitic crystal (as observed by TEM images in Figure 3c,d), which could reduce the diffusion length of the micropores, which would be beneficial to the catalytic activity, catalytic life, the selectivity of epoxy products, and the utilization efficiency of hydrogen peroxide. Meanwhile, the improved hydrophobicity as evaluated by water adsorption testing (Figure 6) was also beneficial to the catalytic performance. However, the recrystallization post-treatment process led to the loss of framework titanium species and the increase of extra-framework titanium species based on the further increased adsorption band at about 260 nm in the DRUV/vis spectra (Figure 4B) compared to sample after acid post-treatment, which was a disadvantage for catalytic performance.
(3) The overall catalytic activity should consider various influence factors comprehensively. The parent nano-zeolite via direct synthesis contained significant amounts of alkali metal ions, displaying a very low catalytic activity. The overall effects were positive to the alkene conversion after removing the alkali metal ions although acid post-treatment, which led to some loss of framework titanium and the decrease of hydrophobicity. The sample after recrystallization and the overall activity was greatly improved.

Through the above research, it could be found that acid treatment was greatly effective for the TS-1 catalyst with high alkali metal content, especially the catalyst that was produced by the preparation process in the presence of high amounts of alkali metal ions in the raw material. However, the improvement by only acid post-treatment was limited. The recrystallization post-treatment method could further improve the catalytic performance of the catalyst. It should be pointed out that parent TS-1 zeolites were directly subjected to recrystallization post-treatment processes without acid washing had less improvement in catalytic performance. Therefore, the combination of acid washing and basic recrystallization two-step post-treatment processes would be an effective strategy to produce the high-performance TS-1 catalyst.

## 4. Conclusions

In summary, high-activity hollow zeolites were stably produced via combined post-treatment strategies of direct-synthesis nano-sized TS-1 zeolites. The key influencing factors during the post-treatment processes to activate TS-1 catalyst were found: (1) removing alkali ions by acids, (2) enhancing the hydrophobic properties via re-crystallization to recover the defects, and (3) improving the utilization efficiency of microporous channels attributed to the nano-cavities and hollow structure. Among above three factors, it was found that the influence of alkali metal ions was more sensitive considering the low content of alkali metal ions and the inevitable contamination in raw materials. It is hoped that these findings will contribute to the stable preparation of high-performance TS-1 catalysts no matter what raw materials were used in both factories and laboratories.

## Figures and Tables

**Table 1:** The porosity properties of synthesized TS-1 samples.

Sample	S_BET_ ^1^ /m^2^ g^−1^	V_micro._ ^2^ /m^3^ g^−1^	S_ext._ ^2^ /m^2^ g^−1^
TS-1-p	467	0.18	78
TS-1-p-ac	469	0.18	96
TS-1-p-ac-re	489	0.18	82

^1^ Specific surface area from Brunauer–Emmett–Teller (BET) method. ^2^ Microporous volume and external specific surface area evaluated by t-plot curves.

**Table 2:** Catalytic oxidation of 1-hexene with H_2_O_2_ by various TS-1 zeolites.

Catalyst	Si/Ti (mol/mol)	Si/K (mol/mol)	Si/Na (mol/mol)	Conversion (%)	Selectivity (%)	TON ^1^ (mol/mol-Ti)	R_init._ ^2^ (×10^−3^/s)	S_H2O2_ ^3^ (%)
TS-1-p	17	58	669	2.1	85.9	5	0.9	28.7
TS-1-p-ac	18	>1000	>1000	7.9	84.7	18	21.1	>99
TS-1-p-ac-re	20	n.d.	n.d.	23.1	98.2	59	49.5	>99
TS-1 [39] ^4^	98	-	-	21.5	100	-	-	84.9
TS-1 [51] ^5^	58	-	-	21.9	-	-	-	-
TS-1 [52] ^6^	40.6	-	-	22.8	95	-	-	93
TS-1 ^7^	45	-	-	24.4	90.4	-	-	80.2

Reaction conditions: Catalyst, 25 mg; 1-hexene, 5 mmol; H_2_O_2_ (30 wt.% in water), 5 mmol; methanol, 5 mL; temperature, 333 K; reaction time, 2 h. ^1^ Turnover number. ^2^ Initial reaction rate per Ti calculated based on the conversion at 10 min. ^3^ Selectivity in the use of H_2_O_2_ toward the epoxides and their derivatives. The symbol of n.d. means that the content was not tested. ^4^ Data from reference [39]. ^5^ Data from reference [51]. ^6^ Data from reference [52]. ^7^ Commercial TS-1 purchased from Catalysis Society of Japan [15].

## Data Availability

We would like to share our data upon request from the authors.
